# Luteolin Inhibits the Biofilm Formation and Cytotoxicity of Methicillin-Resistant *Staphylococcus aureus* via Decreasing Bacterial Toxin Synthesis

**DOI:** 10.1155/2022/4476339

**Published:** 2022-05-09

**Authors:** Yixuan Sun, Fengjun Sun, Wei Feng, Qian Wang, Fang Liu, Peiyuan Xia, Xuewen Qiu

**Affiliations:** ^1^Department of Pharmacy, Chongqing General Hospital, University of Chinese Academy of Sciences, Beijing, China; ^2^Department of Pharmacy, The First Affiliated Hospital of Third Military Medical University (Army Medical University), Chongqing, China

## Abstract

Owing to the fact that luteolin has antibacterial activity against *Staphylococcus aureus* (*S. aureus*) and methicillin-resistant *S. aureus* (MRSA), its specific mechanism in MRSA is worthy of investigation, which is the focus of this study. Initially, the collected *S. aureus* strains were treated with luteolin. Then, the minimum inhibitory concentration (MIC) of luteolin against the *S. aureus* strains was measured by the broth microdilution. The growth curves, biofilm formation, and cytotoxicity of treated *S. aureus* were detected using a microplate reader. The live and dead bacteria were evaluated using confocal laser scanning microscopy, the bacterial morphology was observed using scanning electron microscopy, and the *S. aureus* colony-forming unit (CFU) numbers were assessed. The levels of alpha hemolysin (*α*-hemolysin), delta hemolysin (*δ*-hemolysin), and *hlaA* were detected via western blot and RT-PCR. The mortality of mouse model with *S. aureus* systemic infection was analyzed, and the levels of IL-6, IL-8, IL-10, and TNF-*α* were quantitated using ELISA. Concretely, the MIC of luteolin against MRSA N315 was 64 *μ*g/mL. Luteolin at 16 *μ*g/mL did not affect the growth of MRSA N315, but inhibited the biofilm formation and CFU, and promoted the morphological changes and death of MRSA N315. Luteolin decreased the cytotoxicity and the levels of *α*-hemolysin, *δ*-hemolysin, and *hlaA* in MRSA N315, elevated MRSA-reduced mice survival rate, and differentially modulated the inflammatory cytokine levels in MRSA-infected mice. Collectively, luteolin inhibits biofilm formation and cytotoxicity of MRSA via blocking the bacterial toxin synthesis.

## 1. Introduction

*Staphylococcus aureus* (*S. aureus*) is a Gram-positive bacterium that can lead to food poisoning and human infection and is an important opportunistic pathogen causing human and animal diseases [1, 2]. Studies have shown that *S. aureus* can sense and adapt to various environments within the human host and can access the bloodstream of host and cause severe diseases, such as bacteremia, pneumonia, and endocarditis [2, 3]. The mechanism of disease caused by *S. aureus* is particularly complicated [4]. Research evidence has confirmed that the pathogenic mechanism of *S. aureus* includes adhesion to host cells, resistance to bactericidal and soluble effects of phagocytes, evasion of host immune responses, production or release of other active substances, and direct or indirect cell damage and death [5–7]. At present, *S. aureus* infections are associated with elevated mortality, morbidity, health care costs, and hospital stay, and the death caused by *S. aureus* invasive infections has become a serious public health problem [7, 8].

The discovery of penicillin ushered in the era when antibiotics were used for infection treatment, and infectious diseases caused by *S. aureus* were well-controlled [9]. However, with the widespread use of antibiotics, penicillin-resistant *S. aureus* has emerged in clinical practice [10]. Later, the researchers discovered that methicillin can be used to treat penicillin-resistant *S. aureus*, and methicillin, as a semisynthetic penicillin, has been discovered to exert pharmacological effects by anti-*β*-lactamase degradation [11]. However, it is frustrating that methicillin-resistant *S. aureus* (MRSA) was first isolated by the United Kingdom scientists in 1961, just two years after methicillin had been used in clinical treatment, and MRSA infection has become one of the vital pathogens in community infections so far [11]. Therefore, giving birth to new drugs for treating infectious diseases caused by *S. aureus* and MRSA has become one of the urgent problems to be solved.

With the development of modern pharmacology, some herbal medicines and their related bioactive ingredients have been identified to generate antibacterial effects, of which *Daphne genkwa*, *Momordica charantia*, *Verbena officinalis*, and *Magnolia officinalis* have been used in the study of MRSA [12]. Interestingly, luteolin, as a flavonoid, existed in many plants and bore antibacterial activity against *Trueperella pyogenes* [13]. Also, a previous study has reported that luteolin inhibited the activity of *S. aureus* via downregulating the activity of DNA topoisomerase I and II and hindering the protein and nucleic acid synthesis [14]. Furthermore, a study has documented that luteolin repressed the activity of MRSA [15]. However, the mechanism underlying the antibacterial activity of luteolin against MRSA has not been borne out yet.

In this work, we delved into the effect of luteolin on MRSA and corresponding mechanism, so as to provide new clues for the treatment of diseases infected by MRSA.

## 2. Materials and Methods

### 2.1. Ethics Statement

Our study has obtained the approval from the Ethics Committee of Chongqing General Hospital (CGH2020022005), and 17 adult inpatients with positive blood cultures for *S. aureus* participated in the study, who signed the written informed consent. Meanwhile, all animal experiments were authorized by the Committee of Experimental Animals of Nanfang Hospital (NH2020022005) and performed in accordance with the guidelines of the China Council on Animal Care and Use.

### 2.2. Bacterial Strain Culture and Treatment

Referring to previous experimental methods, we isolated and collected 17 *S. aureus* strains from inpatients [16]; and meanwhile, *S. aureus* ATCC29213 (B81288) and ATCC25923 (MZTPS08) were collected from Mingzhou Biological Technology Co., Ltd. (Ningbo, China). MRSA N315, MRSA N252, and MRSA Mu50 were obtained from Chongqing General Hospital. Then, all bacterial strains were cultured in nutrient agar (WJ6363, bwsm, Beijing, China) with the help of a bacteriological incubator (XY-DR, Xin Yi Instruments and Meters Co., Ltd., Shanghai, China).

In this test, the colony-forming unit (CFU) numbers of *S. aureus* strains were adjusted to 10^5^ CFU/mL and *S. aureus* strains were grown in 6-well plates. Luteolin (L107328), vancomycin (V301569), oxacillin (O114319), azithromycin (A134451), and clindamycin (C274627) were purchased from Aladdin (Shanghai, China), different concentrations (0.5, 1, 2, 4, 8, 16, 32, 64, 128, 256, 512, and 1024 *μ*g/mL) of which were applied to treat *S. aureus* strains for 24 hours. Afterward, treated *S. aureus* strains were collected, and the minimum inhibitory concentration (MIC) was detected. In another test, MRSA N315 and MRSA Mu50 were treated with different concentrations (8, 16, 32, and 64 *μ*g/mL) of luteolin for 24 hours, and were then collected for subsequent studies.

### 2.3. Determination of the MIC and Growth Curves

As previously described [17], the MIC of luteolin, vancomycin, oxacillin, azithromycin, and clindamycin was measured by the broth microdilution according to the Clinical and Laboratory Standards Institute protocol. *S. aureus* strains were treated as previously mentioned; then, the absorbance (at 600 nm) of the wells was detected by a microplate reader (Multiskan SkyHigh, Thermo Fisher Scientific, Waltham, Massachusetts, USA), and the MIC was analyzed.

In line with previous description [18], the bacterial growth curve was analyzed. Briefly, MRSA N315 was maintained in nutrient agar and treated with luteolin at different concentrations (8, 16, 32, and 64 *μ*g/mL) for 24 hours, and the optical density (OD) values (at 600 nm) for isolated strains were detected using a microplate reader at every hour, after which the growth curves were analyzed.

### 2.4. Determination of Biofilm Formation

As previously mentioned [19], the biofilm formation was evaluated. Simply put, MRSA N315 was cultured in nutrient agar supplemented with 0.5% glucose (15023021, ThermoFisher Scientific, Waltham, Massachusetts, USA) in the absence or presence of luteolin at different concentrations (8, 16, 32, and 64 *μ*g/mL) for 24 hours. Thereafter, MRSA N315 was washed with phosphate buffered saline (PBS, P301981, Aladdin, Shanghai, China), fixed using methanol (R40121, ThermoFisher Scientific, Waltham, Massachusetts, USA), then stained with crystal violet solution (0.5%; C299450, Aladdin, Shanghai, China) for 15 minutes(min), and finally washed with distilled water. After drying, the biofilms were solubilized in 33% of glacial acetic acid (A116170, Aladdin, Shanghai, China). Then, the OD values (at 590 nm) were detected by a microplate reader and recorded.

### 2.5. Confocal Laser Scanning Microscopy

Referring to a literature [20], the live and dead bacteria were evaluated using the Live and Dead Bacterial Staining Kit (https://www.qcbio.com/html/45363.htm; 40274ES60, Qcbio Science & Technologies Co., Ltd, Shanghai, China) and confocal laser scanning microscopy (FLUOVIEW FV3000, Olympus, Tokyo, Japan). MRSA N315 was treated with 16 *μ*g/mL of luteolin for 24 hours. Initially, 1 volume of DMAO solution and 2 volumes of EthD-III solution were mixed in a microcentrifuge tube, with the addition of 8 volumes of 0.85% NaCl solution. Later, 100 *μ*L of MRSA N315 suspension was stained with 1 *μ*L of mixed dye solution for 15 min in the dark. Then, 5 *μ*L of the stained bacterial suspension was dropped onto a glass slide (P3963, Aladdin, Shanghai, China). Finally, live (green fluorescence) and dead (red fluorescence) bacteria were observed under a confocal laser scanning microscope.

### 2.6. Determination of CFU

Based on a previous study [21], the CFU numbers of luteolin-treated *S. aureus* were evaluated. MRSA N315 was maintained in Luria-Bertani broth and treated with 1/8 MIC (8 *μ*g/mL), 1/4 MIC (16 *μ*g/mL), or 1/2 MIC (32 *μ*g/mL) of luteolin at 37°C for 24 h, followed by the assessment of *S. aureus* CFU numbers.

### 2.7. Scanning Electron Microscopy

In this test, we observed the bacterial morphology using scanning electron microscopy (JCM7000, JEOL, Tokyo, Japan) as previously described [22]. The MRSA N315 bacterial cells were treated with 16 *μ*g/mL of luteolin at 37°C for 24 hours. After that, bacterial cells were collected, washed using sterilized PBS, then fixed with 2.5% of glutaraldehyde (G105907, Aladdin, Shanghai, China) at 4°C overnight, and treated with 1% of osmium tetroxide (3125103, Qifa Experimental Reagents Co., Ltd., Shanghai, China) at room temperature for 1 hour. Next, the bacterial morphology was observed and photographed (scale bar = 1 *μ*m) under a scanning electron microscope.

### 2.8. Cell Counting Kit (CCK)-8 Assay

The cytotoxicity of MRSA N315 bacteria was detected through CCK-8 assay [23]. Human bronchial epithelial cells (HBEs; CL-0346) were purchased from Procell (Wuhan, China) and grown in the specific cell medium (CM-0346, Procell, Wuhan, China). MRSA N315 bacteria were treated with different concentrations (8, 16, or 32 *μ*g/mL) of luteolin for 24 hours, and then 10 *μ*L of luteolin-treated MRSA N315 bacteria were used to treat HBEs (5000 cells/100 *μ*L) for 24 hours. Afterward, 10 *μ*L of CCK-8 solution was added into each well, and the cells were cultured for 1 hour. Later, the OD values (at 450 nm) were detected by a microplate reader and recorded.

### 2.9. Western Blot Analysis of Alpha Hemolysin (*α*-Hemolysin) and Delta Hemolysin (*δ*-Hemolysin) in MRSA N315 Bacterial

The expressions of alpha hemolysin (*α*-hemolysin) and delta hemolysin (*δ*-hemolysin) in MRSA N315 bacteria were measured through a western blot assay. First of all, MRSA N315 bacteria were treated with 8, 16, or 32 *μ*g/mL of luteolin as delineated above. Then, the protein sample was extracted from MRSA N315 bacteria with the help of a Bacterial Protein Extraction Kit (C600596, Sangon Biotech, Shanghai, China), and the protein concentration was detected utilizing the BCA Protein Assay Kit (C503021, Sangon Biotech, Shanghai, China). Thereafter, SDS-PAGE gel was prepared using the SDS-PAGE Gel Quick Preparation Kit (P0012AC, Beyotime Shanghai, China) to electrophorese 20 *μ*L of the protein sample. Then, the separated protein was transferred onto the PVDF membrane (F019532, Sangon Biotech, Shanghai, China), after which the membrane was blocked by Rapid Block Buff (C500054, Sangon Biotech, Shanghai, China) at room temperature for 1 hour. Subsequently, the membrane was incubated with primary antibodies against *a*-hemolysin (1 : 1000; ab190467, Abcam, Cambridge, UK) and *d*-hemolysin (1 : 1000; PA5-116227, ThermoFisher Scientific, Waltham, Massachusetts, USA) at 4°C overnight, followed by the culture with diluted secondary antibodies (1 : 5000; ab6789, ab205718, Abcam, Cambridge, UK) at room temperature for 1 hour. Next, Western Wash Buffer (P0023C3) and BeyoECL Plus (P0018M) ordered from Beyotime (Shanghai, China) were used to wash and treat the PVDF membrane, after which results were analyzed by the western blot system (FluorChem M, Alpha Innotech, San Francisco, California, USA).

### 2.10. Real-Time PCR (RT-PCR) Analysis of the *hlaA* Expression

In this assay, the Bacteria Total RNA Isolation Kit (B518625) was obtained from Sangon Biotech (Shanghai, China) and applied to isolate a total RNA sample from treated MRSA N315 bacteria, and then the concentration of RNA sample was detected using the spectrophotometer (ND-ONE-W, ThermoFisher Scientific, Waltham, Massachusetts, USA). For the RT-PCR analysis of *hlaA*, the RT-PCR reaction mix solution (including Abstract Taq, one-step RT-PCR buffer, solution I, *hlaA* primer, RNA, and RNase-free H_2_O) was prepared with the help of one-Step RT-PCR Mix (B110025, Sangon Biotech, Shanghai, China), and then was performed on the RT-PCR system (ABI7700, Applied Biosystems, Carlsbad, California, USA). Ultimately, the *hlaA* gene expression was analyzed using the 2^−ΔΔct^ method, and 16S rRNA gene (16S) was used as an endogenous control [24]. The sequence of primers of *hlaA and* 16S is itemized in Table 1.

### 2.11. Mouse Model for *S. aureus* Systemic Infection

 C57BL/6J female mice (6–8 weeks old, *n* = 60) were obtained from ALF Biotechnology Co., Ltd. (Nanjing, China) and housed in specific conditions (12 hour/12 hour light/dark cycle, 22°C, and 55% humidity) with free access to water and food. Next, 60 mice were divided into the following three groups: Mu50 group (*n* = 20), Mu50+ pre-Luteolin group (*n* = 20), and Mu50+ Luteolin group (*n* = 20). In accordance with a former illustration [25], in the Mu50 group, MRSA Mu50 was cultured overnight and diluted with nutrient agar at 1 : 100, then incubated to reach an OD value between 0.45 and 0.6, and washed with PBS, after which the mice were infected with Mu50 (5 ×10^7^, CFU/100 *μ*L) through retro-orbital injection. Similarly, in the Mu50+ pre-luteolin group, mice were infected with luteolin (16 *μ*g/mL)-pretreated Mu50 (5 × 10^7^, CFU/100 *μ*L). Besides, in the Mu50+ luteolin group, mice were infected with Mu50 (5 × 10^7^, CFU/100 *μ*L) and then intraperitoneally injected with luteolin (10 *μ*g/kg) [26]. Following the abovementioned processes, mouse serum samples were collected, and then a survival analysis regarding the infected mice was carried out. Finally, mice were anesthetized using 3% of isoflurane (1349003, Merck, St. Louis, Missouri, USA) and euthanized via cervical dislocation.

### 2.12. ELISA Analysis of Inflammatory Factor Levels

After mice were infected with *S. aureus*, mouse serum samples were collected at the 30 min, 60 min, 90 min, and 120 min. Mice IL-6 (MM-0163M2), IL-8 (MM-0123M2), IL-10 (MM-0176M1), and TNF-*α* ELISA Kits (MM-0132M2) were purchased from MEIMIAN (Jiangsu, China). The experimental reagents, experimental samples, and standard solutions were prepared according to the manufacturer's instructions. Then the prepared samples (50 *μ*L) and standard solutions (50 *μ*L) were added into 96-well plates for incubation at 37°C for 30 min. After the 96-well plate was washed by a wash buffer, enzyme-labeled reagent (50 *μ*L) was added into the 96-well plate and incubated at 37°C for another 30 min. Subsequently, the 96-well plate was washed, chromogenic reagents A (50 *μ*L) and chromogenic reagents B (50 *μ*L) were inoculated into the plate for 10-min culture, and then 50 *μ*L of stop reaction solution was added into each well. Finally, the OD value of each well was detected by a microplate reader.

### 2.13. Statistical Analysis

In this study, all statistical analyses were accomplished with Graphpad8.0 software. Measured data were presented as the mean ± standard deviation, multiple groups were compared by one-way analysis of variance (ANOVA), and *P* < 0.05 was considered as statistically significant.

## 3. Results

### 3.1. Luteolin Inhibited the Biofilm Formation Ability, Reduced CFU, and Promoted the Morphological Changes of MRSA

**Figure 1:**
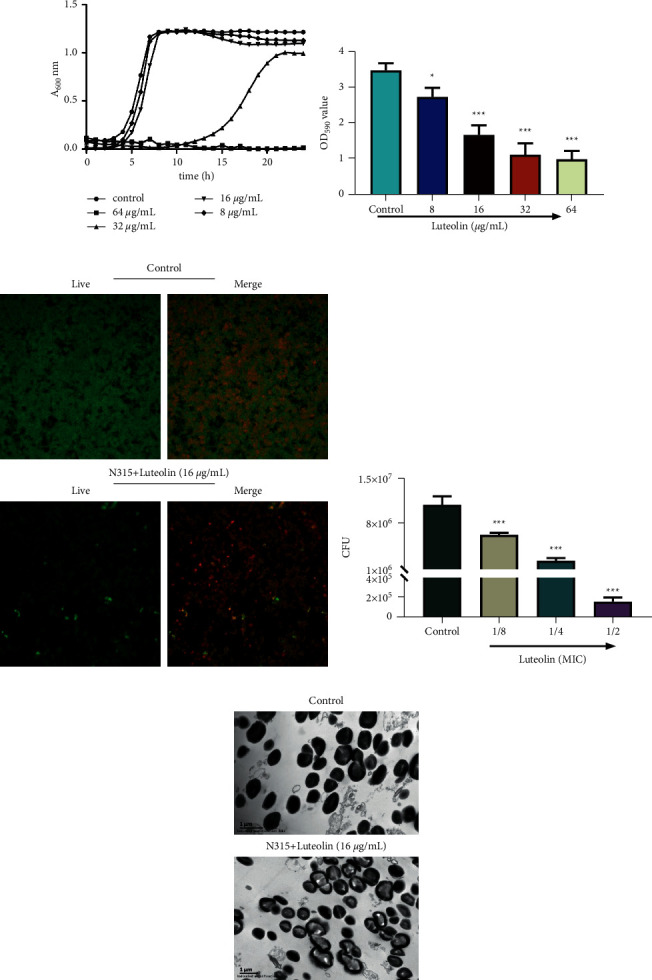
Luteolin inhibited the biofilm formation ability, reduced CFU, and promoted the morphological changes of MRSA. (a)–(e) MRSA N315 was treated with different concentrations (8, 16, 32, and 64 *μ*g/mL) of luteolin, the growth curves (a) and biofilm formation (b) of treated MRSA N315 were assessed by a microplate reader, and then the live and dead bacteria were evaluated using confocal laser scanning microscopy (c), CFU numbers were assessed (d), and the bacterial morphology was observed (scale bar = 1 *μ*m) using scanning electron microscopy (e). ^*∗*^*P* < 0.05, ^*∗∗∗*^*P* < 0.001, vs. Control. (CFU: colony-forming unit, MRSA: methicillin-resistant *Staphylococcus aureus*).

In the light of Table 2, the MIC of luteolin against ATCC29213, ATCC25923, MRSA N315, and *S. aureus* (S7, S9, S13, S16, and S18) was 64 *μ*g/mL, and that against MRSA 252, MU50, and *S. aureus* (S1, S2, S3, S4, S5, S8, S10, S11, S12, S14, S15, and S50) was 128 *μ*g/mL. In subsequent studies, MRSA N315 was treated with different concentrations (8, 16, 32, and 64 *μ*g/mL) of luteolin, and it turned out that luteolin at 16 *μ*g/mL did not impact the growth of MRSA N315 (Figure 1(a)). Luteolin (8, 16, 32, and 64 *μ*g/mL) prominently hampered the biofilm formation of MRSA N315 in a dose-dependent manner (Figure 1(b), *P* < 0.05). In following research studies, MRSA N315 was treated with 16 *μ*g/mL of luteolin. As a result, the observation through a confocal laser scanning microscope on the live (green fluorescence) and dead (red fluorescence) bacteria confirmed that 16 *μ*g/mL of luteolin-induced MRSA N315 death (Figure 1(c)). Likewise, luteolin clearly decreased the CFU of MRSA N315 (Figure 1(d), *P* < 0.001). Besides, it can be observed that the untreated bacterial cell presented intact wall morphology and smooth edge, while MRSA N315 bacterial cell treated with luteolin (16 *μ*g/mL) possessed a sparse wall in light color and with a blurred cell membrane boundary, manifesting that luteolin can regulate the synthesis or depolymerization of MRSA N315 bacterial wall (Figure 1(e)). These data demonstrated that luteolin dampened the biofilm formation ability, diminished CFU, and promoted morphological changes in MRSA.

### 3.2. Luteolin Attenuated the Cytotoxicity of MRSA and Downregulated Hemolysin and *hlaA* Expressions in MRSA

**Figure 2:**
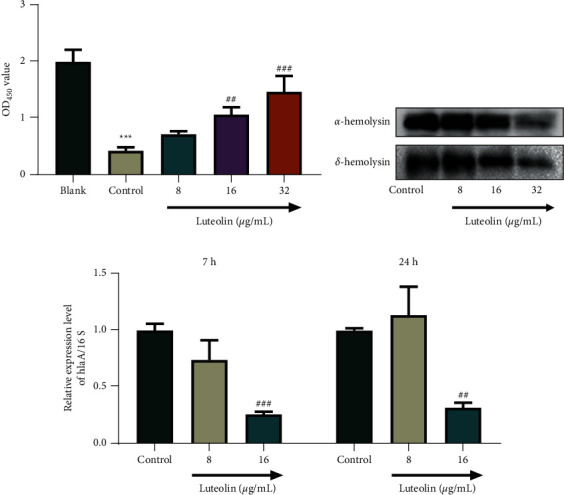
Luteolin decreased the cytotoxicity of MRSA, and downregulated the expression of the hemolysin and *hlaA* genes in MRSA (a) Human bronchial epithelial cells were infected with luteolin-treated MRSA N315, and then the viability of human bronchial epithelial cells was measured using the CCK-8 assay. (b) Expressions of *α*-hemolysin and *δ*-hemolysin in luteolin-treated MRSA N315 were evaluated by western blot. (c) Level of *hlaA* in luteolin-treated MRSA N315 was detected using RT-PCR, and 16S was used as an endogenous control. ^*∗∗∗*^*P* < 0.001, vs. Blank; ^##^*P* < 0.01, ^###^*P* < 0.001 vs. Control. (MRSA: methicillin-resistant *Staphylococcus aureus*, CCK-8: Cell Counting Kit-8, 16S: 16S rRNA, *a*-hemolysin: alpha hemolysin, *d*-hemolysin: delta hemolysin, RT-PCR: Real-Time PCR).

The cytotoxicity of MRSA N315 was determined by the CCK-8 assay; the results of which exhibited that MRSA N315 obviously repressed the viability of HBE cells (Figure 2(a), *P* < 0.001), while luteolin offset the inhibitory effect of MRSA N315 (Figure 2(a), *P* < 0.01). Relevant studies have proved that *a*-hemolysin has cytotoxic effects and *d*-hemolysin can induce cell lysis [27, 28]. In this work, our results indicated that luteolin dwindled the expressions of *a*-hemolysin and *d*-hemolysin in MRSA N315 (Figure 2(b)) and diminished the level of *hlaA* in MRSA N315, when compared with that in the control (Figure 2(c), *P* < 0.001). These findings further verified that luteolin made impacts upon inhibiting MRSA N315 cytotoxicity.

### 3.3. Luteolin Increased the MRSA-Decreased Mice Survival Rate and Regulated the Inflammatory Cytokine Levels in MRSA-Infected Mice

**Figure 3:**
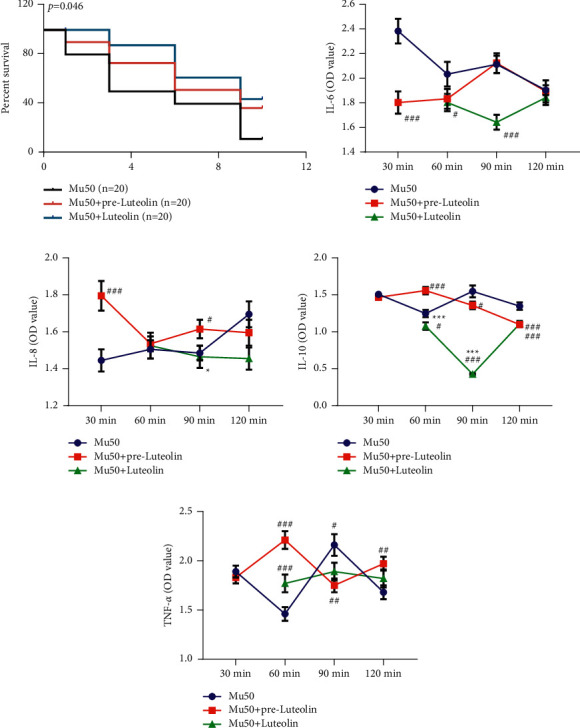
Luteolin increased the MRSA-decreased mice survival rate, and regulated the inflammatory cytokine levels in MRSA-infected mice. (a–e) Mice were infected with MRSA Mu50 or luteolin (16 *μ*g/mL)-pretreated MRSA Mu50, or treated with luteolin (10 *μ*g/kg) by intraperitoneal injection, and serum samples of mice were collected at the 30 min, 60 min, 90 min, and 120 min. Then, the survival rate of infected mice was analyzed (a), and the levels of IL-6 (b), IL-8 (c), IL-10 (d), and TNF-*α* (e) were detected using ELISA. ^*∗*^*P* < 0.05, ^*∗∗∗*^*P* < 0.001 vs. Mu50+ pre-Luteolin; ^#^*P* < 0.05, ^##^*P* < 0.01, ^###^*P* < 0.001, vs. Mu50. (MRSA: methicillin-resistant *Staphylococcus aureus*, IL-6: interleukin 6, IL-8: interleukin 8, IL-10: interleukin 10, and TNF-*α*: Necrosis Factor alpha, ELISA: enzyme-linked immunosorbent assay).

From Figure 3(a), it can be observed that the survival rate of mice was lessened in the Mu50 group but was then augmented in the Mu50+ luteolin group. Furthermore, serum samples of mice were collected after being treated for 30, 60, 90, and 120 min, and the inflammatory cytokine levels were evaluated in MRSA MU50-infected mice (Figure 3(b)–3(e)). In the mouse model, the discoveries revealed that, after mice were treated for 30 min, the IL-6 level in mice of the Mu50+ pre-luteolin group was remarkably lower than that in mice of the Mu50 group (Figure 3(b), *P* < 0.001). Moreover, after mice were treated for 60 and 90 min, the IL-6 level in mice of the Mu50+ luteolin group was lower than that in mice of the Mu50 group (Figure 3(b), *P* < 0.05). In addition, when compared with the Mu50 group, Mu50+ pre-luteolin group had upregulated IL-8 (Figure 3(c), *P* < 0.05), and the Mu50+ luteolin group possessed downregulated IL-8 (Figure 3(c), *P* < 0.05) and IL-10 (Figure 3(d), *P* < 0.05). Besides, the level of TNF-*α* was elevated (60 min) or reduced (90 min) in the Mu50+ luteolin group, when compared with that in the Mu50 group (Figure 3(e), *P* < 0.05). These data implicated that luteolin increased the MRSA MU50-decreased mice survival rate and modulated the inflammatory cytokine levels in MRSA MU50-infected mice.

## 4. Discussion

According to the existing evidence, *S. aureus* can cause acute *S. aureus* toxemia and food poisoning by producing toxic proteins, which seriously affect human health [29]. Although antibiotics can treat *S. aureus* infections to some extent, the emergence of MRSA greatly reduces the therapeutic effect of antibiotics [29]. Recent research results have shown that some Chinese herbal extracts combined with antibiotics have antibacterial effects against MRSA [12]. Additionally, a recent study proved that both *Vetiveria zizanioides* and *Epaltes divaricata* are bacteriostatic against MRSA strains isolated from patients with soft tissue and skin infections [30]. Qian et al. have demonstrated that luteolin inhibits the activity of *S. aureus* by repressing the protein and nucleic acid synthesis [14]. In this study, we also unraveled that luteolin inhibited the activity of *S. aureus.*

Luteolin, a representative natural flavonoid, is widely distributed in nature [31]. Recent studies have verified that luteolin has various biological activities such as antioxidant, antitumor, antibacterial, and antiviral activities [31–33]. Zhang et al. have pointed out that luteolin suppresses the activity of MRSA via disrupting the MRSA cytoplasmic membrane [34]. Biofilm refers to a community of microorganisms that are attached to a surface, which is instrumental in the process of bacterial infection [35]. Besides, an increasing number of experiments have affirmed that the biofilm formation of bacteria is one of the main causes of chronic infection and also considered as the underlying cause of antimicrobial treatment failure [36, 37]. Studies have reported that many traditional Chinese medicines have the effect of antibacterial biofilm formation, such as *Herba patriniae* and baicalin [38, 39]. Qian et al. have indicated that luteolin presents inhibitory effects on the biofilm formation of *S. aureus* [40]. Initial adhesion is perceived to be the initial phase of bacterial biofilm formation, and aloe-emodin has been found to reduce the initial adhesion of *S. aureus* [41]. This research uncovered that luteolin inhibited the biofilm formation ability, reduced CFU, and promoted the morphological changes of MRSA.

Luteolin is an effective and safe natural antioxidant, appropriate concentrations of which not only has no cytotoxicity to human bronchial epithelial cells but also reduces uropathogenic *Escherichia coli*-induced cytotoxicity in human bladder epithelial cells [42–44]. This study for the first time unveiled that luteolin impaired the MRSA-induced cytotoxicity in HBE cells. In addition, it has been indicated previously that most *S. aureus* strains lead to tissue damage via producing a pore-forming cytotoxin *a*-hemolysin [45]. Similarly, *d*-hemolysin is also a pivotal component of *S. aureus* strain products and plays an important role in traversing the plasma membrane [28, 46]. Besides, *hla* has also been reported to be highly associated with immune system function in human [47], which is located on the short arm of chromosome 6 and is the expression product of the major histocompatibility complex in humans [48]. According to the structure, function, cell distribution, and other factors of gene products, *hla* genes are divided into three categories: *hla*-I genes, *hla*-II genes and some other pseudogenes [49, 50]. *HlaA* belongs to the *hla*-I type gene and is the virulence encoding gene in *S. aureus* [51, 52]. In the present study, we first found that luteolin decreased the cytotoxicity of MRSA and downregulated *a*-hemolysin, *d*-hemolysin, and *hlaA* levels in MRSA. These results signified that luteolin attenuated the cytotoxicity of MRSA by reducing the levels of *a*-hemolysin, *d*-hemolysin, and *hlaA* in MRSA.

In related studies, mice are frequently used as animal experimental models with *S. aureus* infection, and MRSA has been uncovered to increase the ratio of MRSA colony count/lung weight in mouse pneumonia model [53]. Furthermore, the *Pithecellobium clypearia* extract enriched in luteolin and gallic acid has antibacterial activity against MRSA [53]. In the present study, luteolin was discovered to increase the MRSA-decreased mice survival rate. Notably, Pratheeshkumar et al. have demonstrated that luteolin repressed the production of pro-inflammatory cytokines TNF-*α*, IL-6, IL-1*β*, and IL-8 in hexavalent chromium-induced human lung epithelial cells [54]. Also, the levels of IL-6, IL-8, and TNF-*α*, as well as the level of anti-inflammatory cytokine IL-10 were evaluated in this study [55]. The findings of the study exhibited that luteolin differentially modulated the levels of IL-6, IL-8, TNF-*α*, and IL-10A at different time points. Hence, the specific modulatory effect of luteolin on the levels of inflammatory cytokines still needs further exploration.

## 5. Conclusion

In summary, our data uncovered that luteolin inhibits the biofilm formation of MRSA, improves the survival rate, and regulates the inflammation of MRSA-infected mice by decreasing the synthesis of some bacterial toxins. Although the role and mechanism of luteolin in patients with MRSA infection are unknown, our findings clarify the potential therapeutic role of luteolin in combating MRSA-infected diseases.

## Figures and Tables

**Table 1:** All primers in RT-PCR experiments in this study.

ID	Sequence (5′-3′)
*hlaA*-forward	TTGGTGCAAATGTTTC
*hlaA*-reverse	TCACTTTCCAGCCTACT
16S-forward	GCTGCCCTTTGTATTGTC
16S-reverse	AGATGTTGGGTTAAGTCCC

**Table 2:** MIC determination of *Staphylococcus aureus*.

Strains	Luteolin	Vancomycin	Oxacillin	Azithromycin	Clindamycin
29213	64	0.5	2	0.5	4
25923	64	0.5	1	0.5	8
N315	64	2	256	1024	1
252	128	2 (1)	256	＞1024	＞1024
MU50	128	8 (1)	512	＞1024	＞1024
S1	128	2	256	1024	＞1024
S2	128	1	256	＞1024	＞1024
S3	128	4	512	1024	＞1024
S4	128	1	512	1024	＞1024
S5	128	2	512	＞1024	＞1024
S7	64	2	256	＞1024	＞1024
S8	128	2	256	1024	＞1024
S9	64	1	512	＞1024	＞1024
S10	128	1	256	＞1024	＞1024
S11	128	8	256	1024	＞1024
S12	128	1	512	＞1024	＞1024
S13	64	1	512	1024	＞1024
S14	128	4	512	1024	＞1024
S15	128	2	512	＞1024	＞1024
S16	64	1	256	＞1024	＞1024
S18	64	2	256	1024	＞1024
S50	128	1	512	＞1024	＞1024

## Data Availability

The analyzed data sets generated during the study are available from the corresponding author on reasonable request.

## References

[1] Li H., Tang T., Stegger M., Dalsgaard A., Liu T., Leisner J. J. (2021). Characterization of antimicrobial-resistant *Staphylococcus aureus* from retail foods in Beijing, China. *Food Microbiology*.

[2] Crosby H. A., Tiwari N., Kwiecinski J. M. (2020). The *Staphylococcus aureus* ArlRS two-component system regulates virulence factor expression through MgrA. *Molecular Microbiology*.

[3] Chang J., Lee R. E., Lee W. (2020). A pursuit of *Staphylococcus aureus* continues: a role of persister cells. *Archives of Pharmacal Research*.

[4] Kwiecinski J. M., Horswill A. R. (2020). *Staphylococcus aureus* bloodstream infections: pathogenesis and regulatory mechanisms. *Current Opinion in Microbiology*.

[5] Mathelie-Guinlet M., Viela F., Alfeo M. J., Pietrocola G., Speziale P., Dufrene Y. F. (2020). Single-molecule analysis demonstrates stress-enhanced binding between *Staphylococcus aureus* surface protein IsdB and host cell integrins. *Nano Letters*.

[6] Flannagan R. S., Heinrichs D. E. (2020). Macrophage-driven nutrient delivery to phagosomal *Staphylococcus aureus* supports bacterial growth. *EMBO Reports*.

[7] Miller L. S., Fowler V. G., Shukla S. K., Rose W. E., Proctor R. A. (2020). Development of a vaccine against *Staphylococcus aureus* invasive infections: evidence based on human immunity, genetics and bacterial evasion mechanisms. *FEMS Microbiology Reviews*.

[8] Sharara S. L., Maragakis L. L., Cosgrove S. E. (2021). Decolonization of *Staphylococcus aureus*. *Infectious Disease Clinics of North America*.

[9] Beier F. (2016). [Treatment of penicillin-sensitive *staphylococcus aureus*]. *Deutsche medizinische Wochenschrift*.

[10] Aspiroz C., Mama O. M., Martinez-Alvarez R. M., Ruiz-Ripa L., Ceballos S., Torres C. (2020). Penicillin-susceptible *Staphylococcus aureus* bacteremia: epidemiological and clinical relevance. Possible therapeutic implications. *Enfermedades Infecciosas y Microbiología Clínica*.

[11] Palavecino E. (2004). Community-acquired methicillin-resistant *Staphylococcus aureus* infections. *Clinics in Laboratory Medicine*.

[12] Kuok C. F., Hoi S. O., Hoi C. F. (2017). Synergistic antibacterial effects of herbal extracts and antibiotics on methicillin-resistant *Staphylococcus aureus*: a computational and experimental study. *Experimental Biology and Medicine*.

[13] Guo Y., Liu Y., Zhang Z. (2020). The antibacterial activity and mechanism of action of luteolin against Trueperella pyogenes. *Infection and Drug Resistance*.

[14] Wang Q., Xie M. (2010). [Antibacterial activity and mechanism of luteolin on *Staphylococcus aureus*]. *Weishengwu Xuebao*.

[15] Su Y., Ma L., Wen Y., Wang H., Zhang S. (2014). Studies of the in vitro antibacterial activities of several polyphenols against clinical isolates of methicillin-resistant *Staphylococcus aureus*. *Molecules*.

[16] Greenberg J. A., Hrusch C. L., Jaffery M. R. (2018). Distinct T-helper cell responses to *Staphylococcus aureus* bacteremia reflect immunologic comorbidities and correlate with mortality. *Critical Care*.

[17] Ucak S., Sudagidan M., Borsa B. A., Mansuroglu B., Ozalp V. C. (2020). Inhibitory effects of aptamer targeted teicoplanin encapsulated PLGA nanoparticles for *Staphylococcus aureus* strains. *World Journal of Microbiology and Biotechnology*.

[18] Zheng X., Fang R., Wang C. (2021). Resistance profiles and biological characteristics of rifampicin-resistant *Staphylococcus aureus* small-colony variants. *Infection and Drug Resistance*.

[19] Jin Y., Guo Y., Zhan Q., Shang Y., Qu D., Yu F. (2020). Subinhibitory concentrations of mupirocin stimulate *Staphylococcus aureus* biofilm formation by upregulating cidA. *Antimicrobial Agents and Chemotherapy*.

[20] Li B., Li X., Lin H., Zhou Y. (2018). Curcumin as a promising antibacterial agent: effects on metabolism and biofilm formation in S. Mutans. *BioMed Research International*.

[21] Prajsnar T. K., Serba J. J., Dekker B. M. (2021). The autophagic response to *Staphylococcus aureus* provides an intracellular niche in neutrophils. *Autophagy*.

[22] Saddiq A. A., Mohamed A. M. (2019). Susceptibility assessment of methicillin-resistant *Staphylococcus aureus* strains to lepidium sativum extract. *Dose Response*.

[23] Ye P., Wei S., Luo C., Wang Q., Li A., Wei F. (2020). Long-term effect against methicillin-resistant *Staphylococcus aureus* of emodin released from coaxial electrospinning nanofiber membranes with a biphasic profile. *Biomolecules*.

[24] Sabersheikh S., Saunders N. A. (2004). Quantification of virulence-associated gene transcripts in epidemic methicillin resistant *Staphylococcus aureus* by real-time PCR. *Molecular and Cellular Probes*.

[25] Krishack P. A., Louviere T. J., Decker T. S. (2019). Protection against *Staphylococcus aureus* bacteremia-induced mortality depends on ILC2s and eosinophils. *JCI Insight*.

[26] Wu B., Song H., Fan M. (2020). Luteolin attenuates sepsisinduced myocardial injury by enhancing autophagy in mice. *International Journal of Molecular Medicine*.

[27] Tang F., Li L., Meng X. M. (2019). Inhibition of alpha-hemolysin expression by resveratrol attenuates *Staphylococcus aureus* virulence. *Microbial Pathogenesis*.

[28] Verdon J., Girardin N., Lacombe C., Berjeaud J. M., Hechard Y. (2009). *δ*-hemolysin, an update on a membrane-interacting peptide. *Peptides*.

[29] Algammal A. M., Hetta H. F., Elkelish A. (2020). Methicillin-resistant *Staphylococcus aureus* (MRSA): one health perspective approach to the bacterium epidemiology, virulence factors, antibiotic-resistance, and zoonotic impact. *Infection and Drug Resistance*.

[30] Rathnayake H., De Zoysa M. H. N., Hewawasam R. P., Gaya Bandara Wijayaratne W. M. D. (2020). Comparison of in vitro antibacterial activity of Epaltes divaricata and Vetiveria zizanioides against methicillin-resistant *Staphylococcus aureus*. *Scientific*.

[31] Imran M., Rauf A., Abu-Izneid T. (2019). Luteolin, a flavonoid, as an anticancer agent: a review. *Biomedicine & Pharmacotherapy*.

[32] Kovacs D., Karancsi Z., Farkas O., Jerzsele A. (2020). Antioxidant activity of flavonoids in LPS-treated IPEC-J2 porcine intestinal epithelial cells and their antibacterial effect against bacteria of swine origin. *Antioxidants*.

[33] Fan W., Qian S., Qian P., Li X. (2016). Antiviral activity of luteolin against Japanese encephalitis virus. *Virus Research*.

[34] Zhang T., Qiu Y., Luo Q. (2018). The mechanism by which luteolin disrupts the cytoplasmic membrane of methicillin-resistant *Staphylococcus aureus*. *Journal of Physical Chemistry B*.

[35] Rabin N., Zheng Y., Opoku-Temeng C., Du Y., Bonsu E., Sintim H. O. (2015). Biofilm formation mechanisms and targets for developing antibiofilm agents. *Future Medicinal Chemistry*.

[36] Tu C., Wang Y., Yi L., Wang Y., Liu B., Gong S. (2019). [Roles of signaling molecules in biofilm formation]. *Sheng Wu Gong Cheng Xue Bao*.

[37] Coenye T., Nelis H. J. (2010). In vitro and in vivo model systems to study microbial biofilm formation. *Journal of Microbiological Methods*.

[38] Fu B., Wu Q., Dang M. (2017). Inhibition of *Pseudomonas aeruginosa* biofilm formation by traditional Chinese medicinal herb Herba patriniae. *BioMed Research International*.

[39] Wang J., Jiao H., Meng J. (2019). Baicalin inhibits biofilm formation and the quorum-sensing system by regulating the MsrA drug efflux pump in Staphylococcus saprophyticus. *Frontiers in Microbiology*.

[40] Qian W., Liu M., Fu Y. (2020). Antimicrobial mechanism of luteolin against *Staphylococcus aureus* and Listeria monocytogenes and its antibiofilm properties. *Microbial Pathogenesis*.

[41] Xiang H., Cao F., Ming D. (2017). Aloe-emodin inhibits *Staphylococcus aureus* biofilms and extracellular protein production at the initial adhesion stage of biofilm development. *Applied Microbiology and Biotechnology*.

[42] Li Y., Shen L., Luo H. (2016). Luteolin ameliorates dextran sulfate sodium-induced colitis in mice possibly through activation of the Nrf2 signaling pathway. *International Immunopharmacology*.

[43] Lee J., Park S. H., Lee J. (2019). Differential effects of luteolin and its glycosides on invasion and apoptosis in MDA-MB-231 triple-negative breast cancer cells. *EXCLI Journal*.

[44] Shen X. F., Ren L. B., Teng Y. (2014). Luteolin decreases the attachment, invasion and cytotoxicity of UPEC in bladder epithelial cells and inhibits UPEC biofilm formation. *Food and Chemical Toxicology*.

[45] Liu S., Zhou X., Li W. (2015). Diosmetin inhibits the expression of alpha-hemolysin in *Staphylococcus aureus*. *Antonie van Leeuwenhoek*.

[46] Tappin M. J., Pastore A., Norton R. S., Freer J. H., Campbell I. D. (1988). High-resolution proton NMR study of the solution structure of .delta.-hemolysin. *Biochemistry*.

[47] Contini P., Murdaca G., Puppo F., Negrini S. (2020). HLA-G expressing immune cells in immune mediated diseases. *Frontiers in Immunology*.

[48] Khaddour K., Hana C. K., Mewawalla P. (2021). *Hematopoietic Stem Cell Transplantation*.

[49] Jordier F., Gras D., De Grandis M. (2020). HLA-H: transcriptional activity and HLA-E mobilization. *Frontiers in Immunology*.

[50] Requena D., Medico A., Chacon R. D., Ramirez M., Marin-Sanchez O. (2020). Identification of novel candidate epitopes on SARS-CoV-2 proteins for south America: a review of hla frequencies by country. *Frontiers in Immunology*.

[51] Tsang J. Y., Ho C. S., Ni Y. B. (2020). Co-expression of HLA-I loci improved prognostication in HER2+ breast cancers. *Cancer Immunology Immunotherapy*.

[52] Jahanshahi A., Zeighami H., Haghi F. (2018). Molecular characterization of methicillin and vancomycin resistant *Staphylococcus aureus* strains isolated from hospitalized patients. *Microbial Drug Resistance*.

[53] Liu C., Huang H., Zhou Q. (2020). Pithecellobium clypearia extract enriched in gallic acid and luteolin has antibacterial activity against MRSA and reduces resistance to erythromycin, ceftriaxone sodium and levofloxacin. *Journal of Applied Microbiology*.

[54] Pratheeshkumar P., Son Y. O., Divya S. P. (2014). Luteolin inhibits Cr(VI)-induced malignant cell transformation of human lung epithelial cells by targeting ROS mediated multiple cell signaling pathways. *Toxicology and Applied Pharmacology*.

[55] Bedke T., Muscate F., Soukou S., Gagliani N., Huber S. (2019). Title: IL-10-producing T cells and their dual functions. *Seminars in Immunology*.

